# Isolation, antimicrobial susceptibility patterns, and risk factors assessment of non-typhoidal *Salmonella* from apparently healthy and diarrheic dogs

**DOI:** 10.1186/s12917-021-03135-x

**Published:** 2022-01-15

**Authors:** Belisa Usmael, Bruk Abraha, Sisay Alemu, Bahar Mummed, Adem Hiko, Abdallahi Abdurehman

**Affiliations:** College of Veterinary Medicine, Haramaya University, P.O. Box 138, Dire Dawa, Ethiopia

**Keywords:** Antimicrobial resistance, Dog, Non-typhoidal *Salmonella*, Prevalence, Risk factors

## Abstract

**Background:**

Dogs are one of the important asymptomatic carriers of antimicrobial resistant and potentially pathogenic strains of *Salmonella*. They can harbor large bacterial load in the intestines and mesenteric lymph nodes which can be shed in their feces with the possibility of transmission to humans. Therefore, a cross-sectional study was conducted with the objectives of estimating the prevalence of non-typhoidal *Salmonella,* assessing the risk factors for dog’s *Salmonella* carriage, and profiling the antimicrobial resistance pattern of *Salmonella* isolates among housed dogs in Harar town, Eastern Ethiopia. A total of 415 rectal swab samples were collected from randomly selected dogs. Samples were examined for non-typhoidal *Salmonella* using standard bacteriologic culture and biochemical tests. The disk diffusion method (Kirby-Bauer test) was employed to evaluate the isolates for their susceptibility against five antimicrobials.

**Results:**

Non-typhoidal *Salmonella* were isolated from 26 (6.3%) of the rectal swab samples, with significantly higher occurrence in diarrheic (15.2%) than non-diarrheic (5.5%) dogs. The risk of *Salmonella* harboring was significantly higher in female dogs than in male dogs (OR = 2.5, *p* = 0.027). Dogs fecal shedding of *Salmonella* was relatively higher in households who used offal as a main feed type for their dogs (23.1%; 95% CI = 5–53.8) than those who used leftover food (10.1%; 95% CI = 5.7–16.1) and practiced mixed feeding system (17%; 95% CI = 7.6–30.8). *Salmonella* isolates showed higher resistance to ampicillin (41.7%), while all isolates were fully susceptible to gentamicin. Moreover, 58.3% of *Salmonella* isolates showed resistance to at least one of the tested antimicrobials. Majorities (72.7%) of the dog owners had no awareness on the risk of zoonotic salmonellosis from dog and all of the respondents use bare hand to clean dog kennel.

**Conclusion:**

Our study reveals the importance of both diarrheic and apparently healthy housed dogs in the harboring and shedding of antimicrobial resistant non-typhoidal *Salmonella*. The risk of non-typhoidal *Salmonella* spread among pet owners is not negligible, especially in households who use offal as main feed type. Therefore, an integrated approach such as: proper dog handling practices; continuous evaluation of antimicrobial resistance; and rational use of antimicrobials in the field of veterinary sector are necessary to tackle the problem.

## Introduction

*Salmonella* is the causative agent of both human and animal salmonellosis. The bacterium causes infections ranging from subclinical carrier state to acute fatal septicemia [1]. It is a potential cause of acute and chronic diarrhea and death in numerous animal species and in human beings [2]. Particularly, salmonellosis in animals is a major concern, because animals can shed *Salmonella* serotypes into the environment without any apparent clinical signs [3]. *Salmonella* is widespread in the environment and commonly found in farm effluents, human sewage and in any material subjected to fecal contamination [4]. Due to considerable geographical and temporal variation in the prevalence of *Salmonella* species in animals and humans, understanding the role of animals in zoonotic transmission is important to monitor salmonellosis [5].

Non-typhoidal *Salmonella* is an important zoonosis worldwide. It is reported that globally an estimated 65–380 million illnesses and 43–88 thousand deaths of human beings were associated with non-typhoidal *S. enterica* from the year 1990 to 2012 [6]. As of 2002, zoonotic *Salmonella* strains such as *S. typhimurium*, S. Heidelberg, and *S. enteritidis* accounts for 17, 11, and 9% of *Salmonella* sourced from non-human subjects [7]. One of the sources for human salmonellosis is feces of pet dogs [8] and there have been reports on transmission of *Salmonella* from dogs to humans [9, 10]. It was reported that dogs can harbor large bacterial load (10^2^–10^6^ per 100 g of feces) in their intestine, which can be shed in their feces for several months [11]. Thus, this carriage could be of significant importance to public health as dogs have close contact with family members in households [12].

Different scholars reported antimicrobial resistant *Salmonella* isolates from food samples [13], animals [14, 15] and human [16, 17] in Ethiopia. Due to the emergence and spread of antimicrobial-resistant strains, there is an increasing concern with this pathogen [18]. The concern of antimicrobial resistance is particularly important in developing countries, because of inadequate adherence to prudent use of antimicrobials; unhygienic living conditions; and close contact and sharing of houses between animals and humans [19].

Some reports have shown the occurrence of *Salmonella* in dogs from different parts of the globe. For instance, United States [20, 21], the United Kingdom [22], Thailand [23], Taiwan [24], Turkey [25, 26], and Trinidad [27]. It has been well known for several decades that dogs may carry *Salmonella* species in their intestinal tracts, mainly as an asymptomatic carrier state [9, 28]. Gastrointestinal disease manifested as enterocolitis and endotoxemia can occur and is often associated with fever, vomiting, anorexia, dehydration, and depression [29, 30]. Pet feed preparations play crucial role in the transmission of *Salmonella* among housed dogs, because raw meat-based dogs feed tends to contain significantly higher *Salmonella* spp. than commercial dry feed [31]. Furthermore, it was reported that *Salmonella* was found in 21% commercial raw food diets, representing combinations of raw meat, vegetables, grain, and eggs or fruit [32]. Contamination rates in dry or canned foods are thought to be considerably lower, and *Salmonella* has not been isolated from canned dog food [33].

Studies in Ethiopia showed that majority of livestock owners have the habit of using antimicrobials to treat animal diseases [34, 35]. However, the antimicrobial usage is characterized by shortcomings such as: inability to define the specific purposes of prescribed drug and lack of awareness on the risks of antimicrobial resistance [34]; the use of human preparation for veterinary purposes, inappropriate dosages, incomplete treatment regimens, lack awareness on the recommended withdrawal periods [35]; and limited access to antimicrobial varieties [34, 35]. In Ethiopia, pet dogs are integral part of the society, which is evidenced by household’s dog ownership ranging from 33 to 40.5% in towns [36, 37] and 75.5% in rural communities [37]. Despite the increasing urbanization in major towns of Ethiopia, only few studies have shown the status of *Salmonella* in housed dogs [15, 38, 39]. Moreover, these studies failed to provide detailed information on the risk factors for dog salmonellosis and there is limited information on the antimicrobial susceptibility profiles of clinical isolates. Therefore, the objectives of this study were to estimate the prevalence of non-typhoidal *Salmonella* isolates, to assess the risk factors associated with *Salmonella* occurrence, and to identify antimicrobial susceptibility profiles of the isolates from apparently healthy and diarrheic dogs in Harar town, Eastern Ethiopia.

## Results

### Overall prevalence of *Salmonella* in dogs

From 415 dogs examined, 26 (6.3%) were positive for *Salmonella*. The present study showed that the point estimates for prevalence of *Salmonella* in apparently healthy and diarrheic dogs was 5.5 and 15.2% respectively. Confidence intervals are given in Table 1.

**Table 1:** Prevalence of *Salmonella* based on clinical status of sampled dogs in Harar town

Clinical state	Number of dogs examined	Number positive for *Salmonella*	Prevalence in % (95% CI)
Apparently healthy	382	21	5.5 (3.4–8.3)
Diarrheic	33	5	15.2 (5.1–31.9)
Total	415	26	6.3 (4.1–9.0)

### Prevalence of *Salmonella* in dogs and households among *Kebeles*

The prevalence varied among *kebeles*, in that it was higher in *kebele* 10 (10%) followed by *kebeles* 15 (9.9%), 16 (7.0%), 18 (3.4%), 13 (1.6%), and 17 (0%) (Table 2) but with no significant variation among the *kebeles*. Among the 209 households, *Salmonella* was detected in 12.4% with varied frequencies among the *kebeles* (Table 2). However, *kebele* had no significant association with the occurrence of *Salmonella* both at animal and household levels. Except in *kebele* 17, *Salmonella* positive dogs were found among households in all *kebeles* and the prevalence varied numerically with the highest being in *kebele* 10 (25%) (Table 2).

**Table 2:** Prevalence of *Salmonella* across the studied *kebeles* of Harar town, eastern Ethiopia

*Kebeles*	Total *No.* of dogs examined	Number of dogs positive for *Salmonella* (%)	Total household examined	Number of household positive For *Salmonella* (%)
15	101	10 (9.9)	72	10 (13.9)
16	142	10 (7.0)	66	10 (14.9)
13	61	1 (1.6)	23	1 (4.3)
10	30	3 (10)	12	3 (25)
18	59	2 (3.4)	29	2 (6.9)
17	22	0 (0)	7	0 (0)
Total	415	26 (6.3)	209	26 (12.4)

### Risk factors for *Salmonella* in dogs

As shown in Table 3, *Salmonella* prevalence was significantly higher in female (10.1%) than males (4.3%) and in diarrheic dogs (15.2%) than apparently healthy (5.5%). Female dogs had 2.5 times the odd of shedding *Salmonella* in their feces than male dogs (*P* < 0.05). The odds of *Salmonella* shedding in thin and fat body conditioned dogs were 2.8 and 1.5 times, respectively higher than medium body conditioned once. Meanwhile, dogs fed uncooked preparations had 2.0 times the odds of harboring *Salmonella* than those fed with cooked preparations. However, there was no significant difference with respect to breed, age, feeding, feed treatment, BCS, and educational status of dog owners (Table 3).

**Table 3:** Results of analysis on potential risk factors for *Salmonella* shedding by dogs in Harar town, Eastern Ethiopia

Variables	No. of Animals examined	No. of Animals with *Salmonella* (%)	χ2 value (*p*-value)	Univariable LG analysis	
				Odds ratio (95% CI)	*p*-value
Sex					
Female	139	14 (10.1)	5.158 (0.023)	2.5 (1.1–5.5)	0.027
Male	276	12 (4.3)		*	
Breed					
Local	306	20 (6.5)	0.146 (0.732)	1.2 (0.5–3.1)	0.703
Cross	109	6 (5.5)		*	
Age					
Young	189	11 (5.8)	0.117 (0.732)	*	
Old	226	15 (6.6)		1.2 (0.5–2.6)	0.732
BSC					
Medium	284	14 (4.9)	3.600 (0.135)	*	
Fat	97	8 (8.2)		1.5 (0.4–5.3)	0.543
Thin	34	4 (11.8)		2.8 (0.8–8.3)	0.115
Feeding					
Leftover	288	15 (5.2)	2.596 (0.262)	*	
Offal’s	27	3 (11.1)		1.6 (0.6–4)	0.312
Both	100	8 (8)		1.4 (0.4–5.8)	0.612
Feed Rx					
Uncooked	31	1 (3.2)	0.527 (0.404)	2.0 (0.3–1.6)	0.478
Mixed	384	25 (6.5)		*	
Diarrheic					
No	382	21 (5.5)	4.821 (0.045)	–	–
Yes	33	5 (15.2)		–	–
Educational status:					
Below high school	251	17 (6.8)	0.279 (0.597)	1.3 (0.5–2.9)	0.598
High school and above	164	9 (5.5)		*	

*N*o*.* Number, *LG* Logistic regression, *CI* Confidence Interval, *BCS* Body condition score, *Rx* Treatment

^*^Explanatory variables

In this study, a relatively higher prevalence of *Salmonella* shedding was observed in households who used offal as main feed type for their dogs (23.1%) than those who used leftover food (10.1%) and practiced mixed feeding system (17%) (Table 4).

**Table 4:** Owners’ awareness on the risk of zoonotic transmission of dog *Salmonella* among households of Harar town, Eastern Ethiopia (*n* = 209)

Variable items	Category	No. of HH respondents	No. positive	Prevalence in % (95% CI)	Chi-square (*p* value)
Feed type	Leftover food	149	15	10.1 (5.7–16.1)	3.026 (0.220)
	Offal	13	3	23.1 (5–53.8)	
	Mixed	47	8	17 (7.6–30.8)	
Feed treatment	Uncooked	19	1	5.3 (0.1–26.0)	0.988 (0.320)
	Mixed	190	25	13.2 (8.7–18.8)	
Educational status of dog owners	Below high school	119	17	14.3 (8.5–21.9)	0.864 (0.238)
	High school and above	90	9	10 (4.7–18.1)	
Knowledge on transmission of *Salmonella* to human	Yes	57	10	17.5 (6.1–16.5)	1.874 (0.171)
	No	152	16	10.5 (8.7–29.9)	

*n* Number of households examined, *No.* Number, *HH* Households

### Antimicrobial susceptibility profiles of *Salmonella* isolates

**Fig. 1:**
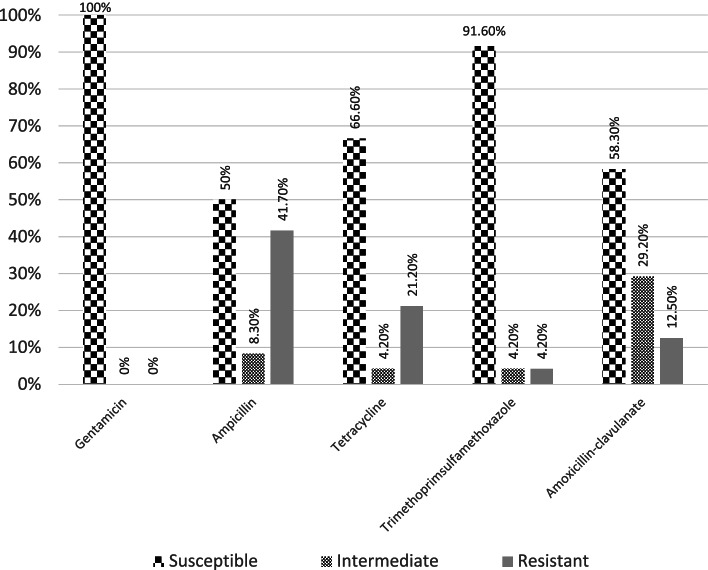
In-vitro antimicrobial susceptibility of the isolated *Salmonella* (*n* = 24)

All (*n* = 24) the tested isolates were susceptible to gentamicin, while varied proportions of resistance were observed against the other tested antimicrobials. Thus, relatively high resistance was observed against ampicillin (41.7%) followed by tetracycline (21.2%), amoxicillin-clavulanate (12.5%), and trimethoprim-sulfamethoxazole (4.2%) (Fig. 1). The control organism was susceptible to all tested antimicrobials.

The study showed that 58.3% of *Salmonella* isolates were resistant to at least one of the tested antimicrobials (Table 5). The dominant isolates were those showed resistance against ampicillin only at a proportion of 20.8%. Meanwhile, from the total *Salmonella* isolates examined, 2 (8.3%) had shown resistance to two antimicrobial classes, with a resistance pattern to ampicillin, tetracycline, and amoxicillin-clavulanate. Moreover, the study revealed that resistant isolates showed similar distribution across the candidate risk factors for dog salmonellosis (Table 6).

**Table 5:** Drug resistance patterns of *Salmonella* isolates (*n* = 24)

Resistant to:	Name of the antimicrobial	Resistant isolates	
		Number	%
None	–	10	41.7
One drug	AMP	5	20.8
	TTC	4	16.6
Two drugs	AMP, TMS	1	4.2
	AMP, AMC	1	4.2
	AMP, TTC	1	4.2
Three drugs	AMP, TTC, AMC	2	8.3
Overall	–	24	100

Key: *n* Number, *AMP* Ampicillin, *TTC* Tetracycline, *TMS* Trimethoprim-sulfamethoxazole, *AMC* Amoxicillin-clavulanate

**Table 6:** Antimicrobial susceptibility profiles of *Salmonella* isolates based on risk categories

Categories	Number (%) of isolates:		
	Resistant to TTC	Resistant to AMP	Susceptible to all
Age			
Young (*n* = 10)	3 (30)	4 (40)	5 (50)
Old (*n* = 14)	4 (28.6)	6 (42.9)	5 (35.7)
Feed			
Leftover food (*n* = 13)	2 (15.9)	4 (30.8)	6 (46.2)
Offal based (*n* = 11)	5 (45.5)	6 (54.5)	4 (36.4)
Sex			
Male (*n* = 11)	3 (27.3)	6 (54.5)	3 (27.3)
Female (*n* = 13)	4 (30.8)	4 (30.8)	7 (53.8)
Breed			
Cross (*n* = 5)	2 (40)	2 (40)	3 (60)
Local (*n* = 19)	5 (26.3)	8 (42.1)	7 (36.8)

*n* Number of Salmonella isolates tested from each variable category, *AMP* Ampicillin, *TTC* Tetracycline

### Dog handling practices in relation to *Salmonella* control

Practices related to dog handling, feeding, and hygiene had varied among households (Table 7). Thus, it was observed that majority (71.3%) of dog owners used leftover food as dog feed and none of them used commercial diet for the feeding of dog. Regarding feed treatment, majority (90.9%) of the households used occasional cooking of feed. Moreover, it was recorded that majority (72.7%) of the dog owners had no awareness on the risk of zoonotic dog salmonellosis. In addition, all of the owners responded that they used to clean dog’s kennel with bare hands.

**Table 7:** Summary of dog management practices and dog owner’s awareness on the risk of zoonotic transmission of *Salmonella* (*n* = 209)

Variable items	Response	Number of respondents	%
Feed types	Commercial diet	0	0
	Leftover food	149	71.3
	Offal	11	5.3
	Mixed	49	23.4
Feed treatment	Uncooked	19	9.1
	Always cooked	0	0
	Sometimes cooked	190	90.9
House cleaning	Use glove	0	0
	Bare hand	209	100
	No clean	0	0
Water source	Tap water	209	100
	Ground water	0	0
Addition of drug to feed	Yes	0	0
	No	209	100
Knowledge on transmission of *Salmonella* to human	Yes	57	27.3
	No	152	72.7

*n* Number of households examined

## Discussion

Our study has focused on prevalence study for *Salmonella* carriage in apparently healthy and diarrheic dogs based on bacteriologic culture and biochemical identification. In addition, an invitro antimicrobial test was conducted using disc diffusion method to observe the resistance profiles of *Salmonella* isolates against five antimicrobials used in the veterinary as well as human medicine. The study also attempts to elucidate the potential risks for the transmission of salmonellosis in dogs as well as humans using a prepared questionnaire format.

Our study showed that the fecal shedding of *Salmonella* among pet dogs located in Harar town of eastern Ethiopia was 6.3%, in which significantly higher prevalence was recorded in diarrheic dogs (15.2%) as compared to the apparently healthy once (5.5%). This finding is within the range of 0 to 44% subclinical carriage of *Salmonella* in dogs [40]. This higher prevalence of *Salmonella* in diarrheic dogs is supported by previous findings in different parts of the globe [8, 41–43]. However, authors like Sultan et al. [38] and Zewdu et al. [39] reported that the prevalence did not vary significantly between clinically healthy and diarrheic dogs in Ethiopia.

The sub-clinical shedding of *Salmonella* by housed dogs has been reported from different countries, but the prevalence varies. For instance, overall sub-clinical *Salmonella* shedding in our study (5.5%) is in line with the report of Amadi et al. [44] and Leahy et al. [20] from Grenada (5.6%) and USA (4.9%), respectively. In contrary to our finding, studies showed lower sub-clinical carriage, such as: 0% [45]; 2.3% [46]; 1% [26]; 1.2% [47]; and 0.2% [48] from New Zealand, USA, Turkey, Canada, and United Kingdom, respectively. This indicates the fact that owners in developed countries may be more focused on the importance of hygiene and make use of the available veterinary care for their animals [49]. On the other hand, different authors have reported higher prevalence such as: 20.8% [50]; 10.5% [3]; 43.7% [51]; 13.2% [43]; 11.7% [15]; and 17.1% [38] from USA, Iran, Northeastern Nigeria, Thailand, Addis Ababa, and Holeta town of Ethiopia, respectively. Generally, prevalence is influenced by factors such as pet sanitary practices, feeding habit, difference in public awareness about dog zoonosis, and socioeconomic status of the owners. Despite the above facts, season of study, geographical areas, and diagnostic methods employed might have also accounted for the observed difference as described by Seepersadsingh et al. [27].

In our findings, there was no significance difference between feeding of leftover, offal and both (leftover and offal). But the prevalence is higher in dogs fed on offal (11.1%) as compared to dogs fed on household leftover food (5.2%) and mixed diet (8%). In agreement with the present finding, Finley et al. [52] reported higher fecal shedding of *Salmonella* in dogs fed on raw meat and offal diets. Schotte et al. [53] stated that feeding raw meat and other uncooked diets were risk factors for carriage of *Salmonella* in dogs. PHAC (Public Health Agency of Canada) [54] reported that raw meat and meat products were frequently contaminated with *Salmonella*, and consequently, homemade raw diets were considered as a potential source of *Salmonella*. Freeman et al. [55] observed that the known infection risk to owners is highly relevant when pets are consuming *Salmonella* contaminated feed. Reports from Ethiopia showed that 8.5–13.5% of examined chicken, pork, mutton, and beef harbor different serotypes of *Salmonella* [13, 56]. Other reports indicate that *Salmonella* is prevalent in animals, humans, and food items in different parts of Ethiopia, suggesting that *Salmonella* can be prevalent in dogs [57, 58].

Our study shows that *Salmonella* shedding was significantly higher in female than male dogs. However, Jajere et al. [59] from Nigerian reported that male dogs had significantly higher *Salmonella* infection than females. In contrary, previous studies from Taiwan [24], Ontario [42], and Mexico [60] showed insignificant difference among male and female dogs. Similarly, from Ethiopia various authors indicated that the prevalence didn’t vary significantly among sex categories of studied dogs [15, 38, 39]. These disparities might not in fact reflect a real phenomenon, but just statistical variation resulting from confounding factors/variables.

In our findings, there was no significance difference between medium, fat and thin body condition score of the dogs, which is in accordance with the reports of Kiflu et al. [15], Sultan et al. [38], and Zewdu et al. [39], in that insignificant difference in the prevalence of *Salmonella* was recorded between body condition categories of studied dogs. Similarly, our study didn’t show significance difference between age categories of dogs. Furthermore, Sultan et al. [38] and Kiflu et al. [15] reported insignificant difference in the prevalence of *Salmonella* between age groups examined. In contrary to our finding, Núñez-Castro et al. [61] from Mexico reported that dogs under 1 year are more likely to acquire *Salmonella* than dogs older than 1 year, while Zewdu et al. [39] from Ethiopia reported that older dogs harbor more *Salmonella* than younger dogs. Often it is difficult to compare different findings, because different age profiles are seen in different studies and it is confounded by differences in sampled population lifestyles and owners dog caring practices. Literatures generally mentioned that younger animals are more susceptible to most of bacterial infections, mainly due to the immature immune system. However, both young and adult animal can be asymptomatic carriers of *Salmonella* [1, 11, 62].

Our finding shows that all *Salmonella* isolates were susceptible to gentamicin. However, previous antimicrobial resistance studies on dog isolates of *Salmonella* species reported resistance to gentamicin in Taiwan (5%) [24] and Nigeria (35.3%) [8]. This may reflect the fact that gentamicin is not commonly used in veterinary sector in Ethiopia, particularly Harar town (Source: researcher’s personal observations and clinical experiences). Meanwhile, some isolates have shown resistance against ampicillin (41.7%), tetracycline (21.2%), amoxicillin-clavulanate (12.5%), and trimethoprim-sulfamethoxazole (4.2%). This high proportion of resistance against ampicillin and tetracycline might reflect their frequent use in veterinary medications. From Ethiopia, Beyene et al. [63] suggest that high-rate of resistance to oxytetracycline is due to the fact that this drug is the most commonly used antimicrobial agent in animal medications. Similarly, a previous study in Taiwan showed resistant isolates to tetracycline (77.5%) and sulfamethoxazole/trimethoprim (37.5%) [24]. From Nigeria, it was reported that *Salmonella* isolates showed resistance to tetracycline (70.6%), ampicillin (47.1%), and amoxicillin-clavulanic acid (87.6%) [8, 51]. A previous report from Ethiopia indicates that 30.9 and 59.5% of *Salmonella* isolates from dogs showed resistance to ampicillin and tetracycline, respectively [15]. These findings indicate that *Salmonella* drug resistance can vary from country to country and even from one area to another area in the same country. The feeding habits of dogs play an important role in contracting drug resistant strains. For instance, Kiflu et al. [15] from Addis Ababa reported that majority of dog owners in the city used raw animal products to feed their dogs. In relation to this, Bedada and Molla [64] showed that 71.3% of beef obtained from cattle slaughtered in central Ethiopia contained oxytetracycline residues.

Majority of the *Salmonella* isolates (58.3%) were resistant to at least one of the tested antimicrobials. Moreover, 8.3% of the isolates showed resistance against three drug types (i.e., ampicillin, tetracycline, and amoxicillin-clavulanate). This shows that apparently healthy dogs could harbor drug resistant *Salmonella* thereby serving as a source of human infection. A better understanding of the interplay of factors that contribute to the dissemination and establishment of multidrug resistant isolates is necessary.

## Conclusion

Our study revealed that non-typhoidal *Salmonella* occurred at higher frequency in diarrheic than apparently healthy dogs with an occurrence in almost all studied small administration units (*kebeles*). *Salmonella* occurrence was relatively higher in dogs managed at households who used offal as main feed type for their dogs than those who used leftover food and practiced mixed feeding system. Thus, dogs might play a significant role in spreading of the organism to humans as well as other animals. Moreover, the high carriage rate of *Salmonella* isolates resistant to varied antimicrobials used in the medications of humans and animals signals an important threat in both the veterinary and public health sectors as it limits antimicrobial drugs available for the effective control of *Salmonella* infections. Regular investigations on the circulating serotype as well as assessing the multi-drug resistance profiles may assist in controlling the occurrence of zoonotic salmonellosis in areas where large proportion of households use dogs as a pet animal.

## Materials and methods

### Study area

The study was conducted in Harar town, which is located around 9^o^N latitude and 42°E longitude and at a distance of about 526 km East of Addis Ababa, the capital of Ethiopia. Harar town has mean annual temperature of 28 °C [65]. The altitude of the town is 1850 m above sea level and its mean annual rainfall and humidity measures 596 mm and 60.3%, respectively [66]. The total human population of the town was estimated at 125,000 with annual growth rate of 2.6% as of the year 2014 [67].

### Study population and sampling units

The study population was dogs owned by residents of Harar town. Among the 19 *kebeles* (i.e. the smallest administrative units of the town) in Harar town, six were randomly selected. Apparently healthy and diarrheic dogs regardless of age, sex, breed, and dog care practices were included in the study. All dogs included were those who didn’t took any medication with antimicrobial activity for the past 4 weeks prior to sampling.

### Study design

A cross-sectional study was conducted from January 2020 to August 2020 to estimate the prevalence of *Salmonella* from rectal swab sample of dogs in selected *kebeles* of Harar towns, Harari Regional State, eastern Ethiopia. Dogs were sampled through door-to-door visit from households. Invitro-experimental study was employed to identify the antimicrobial susceptibility patterns of *Salmonella* isolates.

### Sample size determination

The sample size was determined using the formula given by Thrusfield [68] by assuming simple random sampling. As there was no previous study on dog salmonellosis, the sample size was determined by assuming 50% expected prevalence; 5% desired absolute precision at 95% confidence interval; and based on the assumption of large dog population existing in the town. Thus, with two missed samples, 382 dogs were sampled. In addition, 33 dogs with signs of salmonellosis (diarrhea and septicemia) encountered during the study period were purposively included in the study. Diarrheic dog was defined as an animal presented by owner with a current problem of diarrhea [68].

$$\mathrm n=\frac{1.96^2\mathrm X\;\mathrm{Pexp}\left(1-\mathrm{Pexp}\right)}{\mathrm d^2}$$

Where n = sample size.

P_exp_ = expected prevalence.

d = desired absolute precision.

### Sample and data collection

Prior to sample collection, individual animal’s history of medication with antimicrobial agents was noted. Then rectal swab sample was collected from each dog after proper restraining with the help of the owner. The samples were placed into a sterile buffered peptone water (HiMedia, India) and transported to Haramaya University Veterinary Microbiology Laboratory in box containing ice packs. Samples were processed for bacterial culture within 12 h of arrival. In addition, questionnaire and observational survey were used to gather data on feeding practices (cooked animal products and mixed [raw meat, cooked animal products and household leftover]) and sampled animal attributes such as sex, breed, body condition, and age.

### Isolation and identification of *Salmonella*

Isolation and identification of *Salmonella* from rectal swab samples were performed according to the procedure recommended by the international standard organization (ISO) for isolation of *Salmonella* [69]. Rectal swab samples were transferred into a tube with 9 ml of buffered peptone water (HiMedia, India), shaken for approximately 2 min and incubated at 37 ± 1 °C for 18 ± 2 h. A portion of the culture (0.1 ml) was transferred into a tube containing 10 ml of selective enrichment liquid media (Rappaport-Vassiliadis, HiMedia, India) and incubated at 42 °C for 24 ± 3 h. Similarly, 1 ml of the culture was transferred to a tube containing 10 ml of tetrathionate broth (Conda S.A., Spain) and incubated at 37 °C for 24 ± 3 h. A loopful of inoculum from each of enrichment cultures was then inoculated on the surface of two different plates, xylose lysine deoxycholate (XLD) agar (Sisco research lab, India) and brilliant green agar (BGA) (HiMedia, India) and then incubated at 37 °C for 24 ± 3 h. For confirmation, presumptive *Salmonella* colonies from both XLD and BGA agar were selected and streaked onto the surface of pre-dried nutrient agar (Oxoid, England) plates and incubated at 37 °C for 24 ± 3 h. Colonies from nutrient agar were tested for catalase, oxidase, and Gram’s reaction. Presumptive isolates were inoculated into the following biochemical test tubes for identification: triple sugar iron (TSI) agar (HiMedia, India), Simmon‟s citrate agar (HiMedia, India), Sulphide Indole Motility (SIM) medium (Sisco research lab, India) and incubated for 24 or 48 h at 37 °C. Colonies producing an alkaline (red) slant with acid (yellow) butt with hydrogen sulphide production (blackening) on TSI, positive for citrate utilization (blue color), and negative for tryptophan utilization (Indole test) (yellow-brown ring), and negative for urea utilization were considered as *Salmonella* [70]. In addition, all of the tested isolates were motile. Positive control isolate/strain was obtained from Ethiopian Public Health Institute (EPHI), Addis Ababa, Ethiopia.

### Antimicrobial susceptibility test of *Salmonella* isolates

Susceptibility of the isolates to five antimicrobials was determined using the disk diffusion method according to the guidelines of Clinical and Laboratory Standards Institute [71]. Briefly, frozen isolates were sub-cultured on tryptic soy agar (Becton, Dickinson and Company, USA) from which 3 to 4 pure colonies were further inoculated in to a tube containing 5 ml of tryptic soy broth (TSB) (Becton, Dickinson and Company, USA). The tubes were then incubated at 37 °C for 4–5 h. The turbidity of each suspension was then adjusted to 0.5 McFarland turbidity standard using sterile saline solution. Sterile cotton swab was dipped and rotated several times and pressed firmly on the inside wall of the tube above the fluid level to remove excess inoculum. It was then spreading on to the entire surface of Mueller-Hinton agar plate (Oxoid, Ltd). The inoculated plates were left at room temperature to for 5–10 min until excess moisture is removed and antimicrobial discs were placed by pressing on the plate with sterile forceps. The plates were then inverted and incubated overnight at 35 °C. Diameters of the zone of inhibition were measured to the nearest millimeter using a plastic transparent ruler. The interpretation of the categories of susceptible, intermediate or resistant was based on the CLSI guidelines [71]. For the purpose of analysis, all readings classified as intermediate were considered as resistant unless indicated. Reference strain of *Salmonella Typhi* ATCC 27853 was used as a quality control. The antimicrobial discs (Sensi-Discs, Becton, Dickinson and Company, Loveton, USA) were amoxicillin + clavulanic acid (20/10 μg), gentamicin (10 μg), tetracycline (30 μg), sulfamethoxazole and trimethoprim (23.75 and 1.25 μg), and ampicillin (10 μg).

### Data management and analysis

All collected data were entered and coded using Microsoft Excel Spreadsheet. Statistical analysis was made using STATA software version 11.0 (STATACORP, 2009). Before analysis, the age of dog was classified in to two group young (less than 2 years) and old (> 2 years). in addition, body condition score was done based on 5 scale (emaciated, thin, ideal (medium), fat, and obese) according to AAHA (American Animal Hospital Association). Descriptive statistics such as frequency and percentage were used to describe the practices, knowledge and awareness in the community regarding the disease. Chi-square, Fisher exact test and logistic regression analyses were used to assess the association of risk factors with the prevalence of *Salmonella*. In all the cases, *P* < 0.05 was considered as significant association.

## Data Availability

The data used to validate the results of this analysis are available from the first and correspondent authors upon reasonable request.

## Abbreviations

BGA: Brilliant Green Agar
TSI: Triple Sugar Iron
XLD: Xylose Lysine Deoxycholate
